# Beyond COP28: Brazil must act to tackle the global climate and biodiversity crisis

**DOI:** 10.1038/s44185-024-00051-9

**Published:** 2024-08-21

**Authors:** Flávia de Figueiredo Machado, Marcela C. N. S. Terra, André Ferreira Rodrigues, Philip M. Fearnside, Luís Fernando Guedes Pinto, Polyanna da Conceição Bispo, Frederico V. Faleiro, André G. Coutinho, André Luis Regolin, Carolina Jaramillo-Giraldo, Fabiano R. Melo, Felipe P. L. Melo, Ima C. G. Vieira, Lara M. Monteiro, Luís G. A. Barboza, Madelaine Venzon, Raísa R. S. Vieira, Rosângela Corrêa, Sheila M. Pessoa, Fernando M. Pelicice

**Affiliations:** 1 Programa de Pós-Graduação em Biodiversidade, Ecologia e Conservação, Núcleo de Estudos Ambientais, Universidade Federal do Tocantins, 77.500-000 Porto Nacional, Tocantins Brazil; 2 Departamento de Engenharia Florestal, Universidade Federal de São João del-Rei, CP 56, 35701-970 Sete Lagoas, Minas Gerais Brazil; 3 Departamento de Engenharia Hidráulica e Recursos Hídricos, Escola de Engenharia, Universidade Federal de Minas Gerais, CP 6627, 31270-901 Belo Horizonte, Minas Gerais Brazil; 4 Instituto Nacional de Pesquisas da Amazônia (INPA), Av. André Araújo, 2936, 69067-375 Manaus, Amazonas Brazil; 5 SOS Mata Atlântica, Av. Paulista, 2073 - Horsa 1 - Conj. 1318, 01311-300, 57.354-540 São Paulo, Brazil; 6 Department of Geography, School of Environment, Education and Development, University of Manchester, Manchester, UK; 7 Biota Projetos e Consultoria Ambiental Ltda, Rua 86-C, 64, Setor Sul, CEP 74083-360 Goiânia, Goiás Brazil; 8 Laboratório de Ecologia Funcional de Comunidades (LABEF), Departamento de Botânica, Setor de Ciências Biológicas, Universidade Federal do Paraná, Curitiba, Brazil; 9 Departamento de Ecologia, Instituto de Ciências Biológicas, Universidade Federal de Goiás - UFG, Goiânia, Goiás Brazil; 10 Empresa de Pesquisa Agropecuária de Minas Gerais (EPAMIG), Vila Gianetti 46, CEP 36570 000 Viçosa, MG Brazil; 11 Departamento de Engenharia Florestal, Universidade Federal de Viçosa, 36570-900 Viçosa, Minas Gerais Brazil; 12 School of Animal, Rural and Environmental Sciences, Nottingham Trent University, Nottingham, UK; 13 Centro de Biociências, Universidade Federal de Pernambuco, Recife, Brazil; 14 Museu Paraense Emilio Goeldi (MPEG), av Magalhães Barata 376, 66040-170 Belém, Pará Brazil; 15 Rubenstein School of Environment and Natural Resources, 81 Carrigan Drive, Burlington, VT 05405 USA; 16 Gund Institute for Environment, University of Vermont, Farrell Hall, 210 Colchester Avenue, Burlington, VT 05405 USA; 17 CIIMAR – Interdisciplinary Centre of Marine and Environmental Research, University of Porto, Research Team of Aquatic Ecotoxicology and One Health (ECOTOX), Matosinhos, Portugal; 18 Department of Populations Study, Laboratory of Ecotoxicology and Ecology, ICBAS, University of Porto, Porto, Portugal; 19 International Institute of Sustainability (IIS), 22460-320 Rio de Janeiro, Brazil; 20 Universidade de Brasília, Faculdade de Educação, Campus Universitário Darcy Ribeiro Asa Norte 70910900, Brasília, Distrito Federal Brasília, Brazil; 21 Expressão Natureza, 75064-020 Anápolis, Goiás Brazil

**Keywords:** Biodiversity, Conservation biology, Ecology, Climate sciences, Scientific community, Social sciences

## Abstract

Extreme weather has made 2023 virtually certain to be the warmest year on record, signaling unprecedented climate and biodiversity crises. Brazil, the world’s most biodiverse country, with two hotspots and complex social and economic layers, has experienced escalating environmental degradation over the past years. Alarming rates of native vegetation loss, wildfires, severe and prolonged droughts, and heatwaves have adversely impacted several Brazilian ecosystems and societies. Despite the country’s decisive role in global carbon neutrality, bridging the gap between Brazil’s discourse on the international stage and its concrete actions at home remains a significant challenge. This correspondence, a collective plea from scientists across various sectors, underscores the urgent imperative for national engagement and commitment to halt and mitigate these crises. We aim to catalyze a robust international public debate, influencing Brazilian decision-makers to chart a concrete sustainable pathway. Aligned with global initiatives, we emphasize the crucial interplay between national and international efforts in combating climate change and the conservation of biodiversity and socio-biodiversity.

Extreme weather events have made 2023 virtually certain to be the warmest year on record. Global droughts, floods, and heatwaves confirm that we are facing a climate crisis^1^, and the Anthropocene’s biodiversity crisis poses unprecedented threats to ecosystems and societies. Despite being the world’s most biodiverse country with two hotspots^2^, Brazil has experienced escalating environmental degradation over the past years, demanding national engagement and commitment to contain the climate and biodiversity crises. The inauguration of President Luiz Inácio Lula da Silva’s third mandate heralded a marked improvement in Brazil’s environmental prospects. While Brazil needs to assume global leadership in confronting the climate and biodiversity crises, a gulf between the country’s discourse on the international stage and its concrete actions at home has impeded this^3^.

Although Brazil’s GHG emissions decreased by 8% in 2022, totaling 2.3 billion metric tons of CO_2_, it was the third-highest year in emissions since 2005, keeping Brazil among the top 10 global emitters. Land use and cover change (responsible for 48% of emissions) have declined due to lower deforestation rates in 2023 in the Amazon (50%) and Atlantic (59%) forests^4^. However, the loss of non-forest vegetation, particularly the Cerrado (central Brazilian savanna), has received less public attention^5^. The annual loss of Cerrado increased 65.9%^4^ from 2018 to 2023. In the same period, vegetation coverage declined by 58.4% in the Pantanal, 19.7% in the Brazilian Pampa, and 18.2% in the Caatinga^4^. In the last 38 years, Brazil has lost 33% of its native vegetation, and non-forest natural vegetation shrank by 9.6 million hectares (a 16% decline). Wildfires are another concern; despite a 1% nationwide decrease in the first half of 2023, wildfires increased in the Amazon by 14% (Fig. 1) and in the Cerrado by 2%. Together, they comprised 98% of Brazil’s burnt area, of which an alarming 84% was in native vegetation. All ecosystems – marine, terrestrial, freshwater, and subterranean—have been negatively affected by human activities in Brazil^1^. Throughout the country, vegetation loss driven by the expansion of cattle ranching and modern agribusiness has aggravated the impact of extreme floods and droughts caused by El Niño^6,7^, intensified by climate change. The Amazon has been facing severe droughts and heatwaves, while prolonged droughts in the Cerrado have negatively impacted the water supply to major Brazilian watersheds^8^. Beyond climate change and habitat loss, other stressors have threatened Brazilian ecosystems^1^, including pollution, invasive species, overexploitation, epidemics, and emerging diseases.

At the November 2023 Conference of the Parties for the UN Climate Convention (COP28), Brazil led climate mitigation pledges, promising a 48% reduction in greenhouse gas emissions by 2025 and 53% by 2030. Beyond COP28’s 1.5 °C target, Brazil is part of the 30 × 30 Biodiversity Framework and the UN Decade on Ecosystem Restoration. However, despite the re-establishment of the Environmental agenda by Brazil’s current Environment Ministry, some of the government entities, such as the Ministry of Transportation and the Ministry of Mines and Energy, promote projects with enormous impacts on biodiversity and climate^9^. Despite the current President desire to rehabilitate Brazil’s environmental leadership, his administration’s contradictions and an openly anti-environmental right-wing Congress pose risks to Brazilian social-ecological commitments. For example, in December 2023, Brazil joined the OPEP+ group of oil and gas-producing countries and auctioned off 602 drilling blocks spanning nine sedimentary basins (21 in the Amazon), threatening 20 indigenous lands, buffer zones, and 15 protected areas. Simultaneously, the National Congress approved a “time benchmark” (*marco temporal*) that hinders the demarcation of indigenous territories, affecting biodiversity and the persistence of 266 ethnic groups, violating human rights outlined in Brazil’s 1988 constitution. In Brazil’s National Congress, where the Agribusiness Front has over 60% of the seats in both houses, various bills are advancing towards approval that would have disastrous consequences for sustainability (e.g., PL 364/19, which would leave 48 million hectares unprotected).

**Fig. 1:**
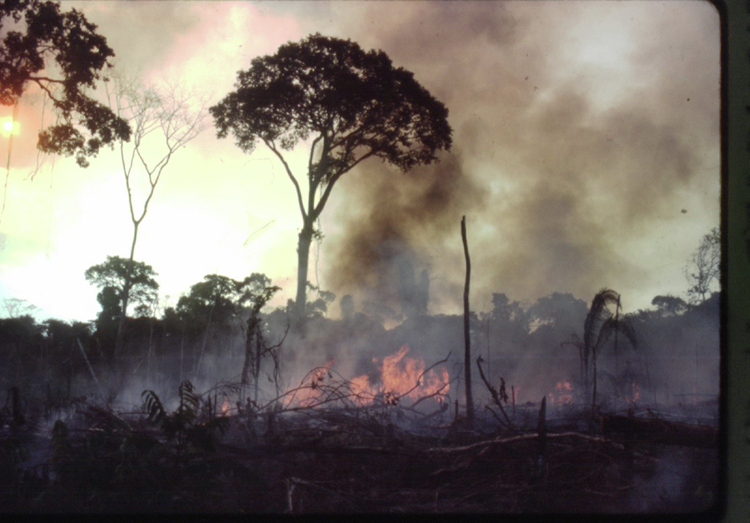
Amazon forest on fires. (Picture: Philip Fearnside). https://amazoniareal.com.br/queimadas-batem-recorde-em-agosto-na-amazonia/.

Urgent needs to combat the climate and biodiversity crises and improve social justice include restoring degraded ecosystems, implementing more protected areas, encouraging good agricultural practices, and achieving zero loss of native vegetation throughout Brazil. The restoration actions also need to be implemented in drier ecosystems, which hold great carbon sequestration potential^5^. The current pace of degradation and losses demands policies to safeguard the remaining vegetation in private, public, traditional communities, and indigenous lands^10^. This is mandatory for Brazil to achieve carbon neutrality and assume global environmental leadership. Brazil’s development needs to be aligned with ongoing initiatives like the Intergovernmental Science-Policy Platform on Biodiversity and Ecosystem Services (IPBES), emphasizing empowering ‘bottom-up’ agendas, reconnecting environmental and social justice, respecting traditional people and communities, and supporting grassroots socio-environmental movements for a state linked to nature. The COP30, which will be held in Brazil, would be an opportune moment to consolidate these actions. Brazilian president’ commitment to ending poverty and addressing social inequalities by integrating climate policies across 23 ministries emphasizes the need for an integrated, articulated, and interdisciplinary approach to deal with both crises, and it demands aligning discourse with practice. Failing this task risks perpetuating the sixth mass extinction, impacting ecosystems and human societies globally.
