# Author Correction: Beyond COP28: Brazil must act to tackle the global climate and biodiversity crisis

**DOI:** 10.1038/s44185-024-00069-z

**Published:** 2024-11-28

**Authors:** Flávia de Figueiredo Machado, Marcela C. N. S. Terra, André Ferreira Rodrigues, Philip M. Fearnside, Luís Fernando Guedes Pinto, Polyanna da Conceição Bispo, Frederico V. Faleiro, André G. Coutinho, André Luis Regolin, Carolina Jaramillo-Giraldo, Fabiano R. Melo, Felipe P. L. Melo, Ima C. G. Vieira, Lara M. Monteiro, Luís G. A. Barboza, Madelaine Venzon, Raísa R. S. Vieira, Rosângela Corrêa, Sheila M. Pessoa, Fernando M. Pelicice

**Affiliations:** 1 Programa de Pós-Graduação em Biodiversidade, Ecologia e Conservação, Núcleo de Estudos Ambientais, Universidade Federal do Tocantins, 77.500-000 Porto Nacional, Tocantins Brazil; 2 Departamento de Engenharia Florestal, Universidade Federal de São João del-Rei, CP 56, 35701-970 Sete Lagoas, Minas Gerais Brazil; 3 Departamento de Engenharia Hidráulica e Recursos Hídricos, Escola de Engenharia, Universidade Federal de Minas Gerais, CP 6627, 31270-901 Belo Horizonte, Minas Gerais Brazil; 4 Instituto Nacional de Pesquisas da Amazônia (INPA), Av. André Araújo, 2936, 69067-375 Manaus, Amazonas Brazil; 5 SOS Mata Atlântica, Av. Paulista, 2073 - Horsa 1 - Conj. 1318, 01311-300, 57.354-540 São Paulo, Brazil; 6 Department of Geography, School of Environment, Education and Development, University of Manchester, Manchester, UK; 7 Biota Projetos e Consultoria Ambiental Ltda, Rua 86-C, 64, Setor Sul, CEP 74083-360 Goiânia, Goiás Brazil; 8 Laboratório de Ecologia Funcional de Comunidades (LABEF), Departamento de Botânica, Setor de Ciências Biológicas, Universidade Federal do Paraná, Curitiba, Brazil; 9 Departamento de Ecologia, Instituto de Ciências Biológicas, Universidade Federal de Goiás - UFG, Goiânia, Goiás Brazil; 10 Empresa de Pesquisa Agropecuária de Minas Gerais (EPAMIG), Vila Gianetti 46, CEP 36570 000 Viçosa, MG Brazil; 11 Departamento de Engenharia Florestal, Universidade Federal de Viçosa, 36570-900 Viçosa, Minas Gerais Brazil; 12 School of Animal, Rural and Environmental Sciences, Nottingham Trent University, Nottingham, UK; 13 Centro de Biociências, Universidade Federal de Pernambuco, Recife, Brazil; 14 Museu Paraense Emilio Goeldi (MPEG), av Magalhães Barata 376, 66040-170 Belém, Pará Brazil; 15 Rubenstein School of Environment and Natural Resources, 81 Carrigan Drive, Burlington, VT 05405 USA; 16 Gund Institute for Environment, University of Vermont, Farrell Hall, 210 Colchester Avenue, Burlington, VT 05405 USA; 17 CIIMAR – Interdisciplinary Centre of Marine and Environmental Research, University of Porto, Research Team of Aquatic Ecotoxicology and One Health (ECOTOX), Matosinhos, Portugal; 18 Department of Populations Study, Laboratory of Ecotoxicology and Ecology, ICBAS, University of Porto, Porto, Portugal; 19 International Institute of Sustainability (IIS), 22460-320 Rio de Janeiro, Brazil; 20 Universidade de Brasília, Faculdade de Educação, Campus Universitário Darcy Ribeiro Asa Norte 70910900, Brasília, Distrito Federal Brasília, Brazil; 21 Expressão Natureza, 75064-020 Anápolis, Goiás Brazil

**Keywords:** Biodiversity, Conservation biology, Ecology, Climate sciences, Scientific community, Social sciences

**Correction to:** *npj Biodiversity* (2024) **3**:19; 10.1038/s44185-024-00051-9, published online 21 August 2024

In this article the affiliation details for Author Polyanna da Conceição Bispo were incorrectly given as ‘^6^Department of Geography, School of Environment, Education and Development, University of Manchester, Manchester, United Kingdom. ^7^Biota Projetos e Consultoria Ambiental Ltda. Rua 86-C, 64, Setor Sul, CEP 74083-360, Goiânia, Goiás, Brasil.’ but should have been ‘^6^Department of Geography, School of Environment, Education and Development, University of Manchester, Manchester, United Kingdom’. The original article has been corrected.

